# Inner speech in the daily lives of people with aphasia

**DOI:** 10.3389/fpsyg.2024.1335425

**Published:** 2024-03-21

**Authors:** Julianne M. Alexander, Tessa Hedrick, Brielle C. Stark

**Affiliations:** ^1^Department of Speech, Language and Hearing Science, Indiana University Bloomington, Bloomington, IN, United States; ^2^Program in Neuroscience, Indiana University Bloomington, Bloomington, IN, United States

**Keywords:** aphasia, inner speech, experience sampling method, survey, language, feasibility

## Abstract

**Introduction:**

This exploratory, preliminary, feasibility study evaluated the extent to which adults with chronic aphasia (*N* = 23) report experiencing inner speech in their daily lives by leveraging experience sampling and survey methodology.

**Methods:**

The presence of inner speech was assessed at 30 time-points and themes of inner speech at three time-points, over the course of three weeks. The relationship of inner speech to aphasia severity, demographic information (age, sex, years post-stroke), and insight into language impairment was evaluated.

**Results:**

There was low attrition (<8%) and high compliance (>94%) for the study procedures, and inner speech was experienced in most sampled instances (>78%). The most common themes of inner speech experience across the weeks were ‘when remembering’, ‘to plan’, and ‘to motivate oneself’. There was no significant relationship identified between inner speech and aphasia severity, insight into language impairment, or demographic information. In conclusion, adults with aphasia tend to report experiencing inner speech often, with some shared themes (e.g., remembering, planning), and use inner speech to explore themes that are uncommon in young adults in other studies (e.g., to talk to themselves about health).

**Discussion:**

High compliance and low attrition suggest design feasibility, and results emphasize the importance of collecting data in age-similar, non-brain-damaged peers as well as in adults with other neurogenic communication disorders to fully understand the experience and use of inner speech in daily life. Clinical implications and future directions are discussed.

## Introduction

### The inner speech phenomenon

Inner speech’s definition varies by discipline, having been investigated under the contexts of psychology, neuroscience, philosophy, and cognitive science. The simplest definitions describe the experience of a “little voice in the head” (Geva et al., 2011a) or “silent self-directed speech” (Vygotsky, 1962). The ConDialInt Model (Grandchamp et al., 2019) envisions inner speech as multidimensional, comprising dimensions of condensation, dialogality, and intentionality. The condensation dimension reflects inner speech’s representation as a purely condensed form (without all acoustic, phonological, and syntactic information) to an expanded form (including articulatory and auditory properties). Indeed, “thinking in pure meaning” (Vygotsky, 1962) would be the extreme of the condensed form of inner speech. The dialogality dimension reflects inner speech’s continuum from monologic (an inner soliloquy) to dialogic (reflecting a back/forth conversation). The intentionality dimension ranges from intentional, as when purposefully rehearsing information or manipulating it silently for later use, to unintentional, as in daydreaming or mind wandering. Recent research suggests that inner speech may be better conceptualized (and empirically studied) by re-envisioning the ConDialInt model (Pratts et al., 2023). Pratts et al. (2023) eliminate “condensation,” as they argue that this dimension is not empirically manipulatable or measurable, and replace “dialogality” with egocentricity, measuring the extent to which inner speech is a recreation of one’s own voice (high egocentricity), or the voice of another individual (low egocentricity). They argue that this more clearly differentiates the distinct types of inner speech (own-voice vs. other-voice) to enable empirical investigation. Pratts et al. (2023) also replace “intentionality” with spontaneity, largely to improve the definition of this aspect of inner speech to represent the fact that spontaneous inner speech may or may not have intent. The spontaneity continuum ranges from highly deliberate inner speech, elicited by explicit task demands (e.g., rhyme judgements), to highly spontaneous inner speech emerging in the absence of cues or clear task demands (e.g., mind wandering). In philosophy literature, researchers disagree as to which components of inner speech are required, theorizing about matters such as how conscious people are of their inner speech, how much phonetic and articulatory information is involved, and how inner speech interacts with other cognitive processes (Langland-Hassan et al., 2018). To summarize, the definitions of inner speech, the rationale behind measuring inner speech, and the methods used to measure inner speech (discussed in more detail later in the Introduction) likely have some bearing on the types of data gathered and their interpretation. Despite the variability of studies, the accumulation of evidence on inner speech from a variety of disciplines, methodologies, and subject groups makes more complete and clear the role of inner speech in cognition and, more broadly, in daily life.

### Frequency and content of inner speech

Inner speech experiences happen variably in young adults (in 0–75% of cases, estimated using experience sampling methods; Heavey and Hurlburt, 2008). Studies using surveys rather than experience sampling, asking more generally about inner speech experience, have identified a majority of young adults as experiencing inner speech (e.g., 68% using Nevada Inner Experience Questionnaire and 67% using the Self-Talk Scale; Brinthaupt et al., 2009; Heavey et al., 2019).

Through surveys such as the General Inner Speech Questionnaire (Racy et al., 2020), inner speech has been shown to vary by context (e.g., using inner speech when driving, alone, or working) and have many different uses (e.g., problem-solving, controlling emotions, or self-evaluating). Through various methodologies in neurotypical populations, inner speech has been closely linked with self-awareness and monitoring (Morin and Everett, 1990; Nooteboom, 2005; Morin, 2011a,b; Perrone-Bertolotti et al., 2014), emotion regulation and assessment (Kross et al., 2014; Racy et al., 2020; Morin and Racy, 2022), and cognitive processes such as problem-solving (Alderson-Day and Fernyhough, 2015; Wallace et al., 2017), planning (Racy et al., 2020; Morin and Racy, 2022), and memory (Racy et al., 2020). In fact, interrupting inner speech disrupted problem-solving abilities in young adults (Wallace et al., 2017), suggesting that inner speech may be critical for solving certain kinds of problems.

### Inner speech in aphasia

Aphasia is a language disorder that affects two million adults in the USA and is most commonly caused by a stroke to the dominant (left) hemisphere (Simmons-Mackie and Cherney, 2018). Typically, aphasia persists into the chronic stage (usually defined as more than six months post-injury) in 20–40% of individuals with acute aphasia (Flowers et al., 2016). In adults with aphasia who have impairments of verbal language, inner speech can still be relatively intact (Geva et al., 2011a; Fama et al., 2017; Stark et al., 2017; Alexander et al., 2023). This is supported by findings from cognitive neuroscience research, demonstrating that inner speech has distinct neural correlates from overt speech (Geva et al., 2011b; Pillay et al., 2014; Fama et al., 2017), and that subjective experiences of inner speech dissociate from experiences of “tip of the tongue” phenomena and “having an idea without a word” (Fama et al., 2019b). That is, inner speech is not simply ‘impoverished’ overt speech. To date, the majority of empirical studies evaluating inner speech in aphasia have leveraged experimental, non-spontaneous, and highly constrained tasks, such as silent rhyme judgment (Geva et al., 2011b; Stark et al., 2017; Geva and Warburton, 2018), as a proxy for inner speech presence. These investigations have highlighted inner speech’s relationship with language functions like phonological working memory and phonological retrieval (Stark et al., 2017; Fama et al., 2019a).

Inner speech is interesting in aphasia not only because of its ability to help identify the source of anomia (word finding impairments, for an overview see Fama and Turkeltaub, 2020), but because many individuals with aphasia anecdotally speak about being ‘able to say a word in their head’ and not aloud, and who anecdotally speak about inner speech during daily life. Stroke survivor Shai Anbar, in *Goddess Aphasia: A Stroke Survivor and his Dual Muse*, wrote that all forms of language are affected by aphasia, including inner speech (Anbar, 2022). Taylor, a neuroanatomist, suffered a left-hemisphere stroke which caused a temporary but nearly complete loss of inner speech immediately post-stroke. She highlights the importance of inner speech in her book *My Stroke of Insight,* describing the lack of inner speech as “the dramatic silence that had taken residency inside my head” (Taylor, 2008, pp. 75–76). Dr. Bolte Taylor stated that the silence affected her self-awareness and sense of self (p. 67), but it is unknown whether this is common to most people living with aphasia. Both Mr. Anbar and Dr. Bolte Taylor describe the loss of inner speech acutely, with some indication that inner speech has the potential to return over the course of stroke recovery. Indeed, inner life and the experience of inner speech may be more rich post-stroke than it was pre-stroke, with recent evidence suggesting that individuals with moderate aphasia subjectively say that they experience inner speech more frequently after their stroke (Alexander et al., 2023). Because inner speech plays a variety of roles for adults without brain damage, the preservation and uses of inner speech by adults with aphasia may constitute an important factor in their recovery and in living well with aphasia.

### Measuring inner speech in daily life

In neurotypical adult research, there has been progress in formalizing models and measurements of inner speech, with a great emphasis on being able to “objectively” measure this phenomenon with experimental control, such as silent reading, rhyming, and homophone judgments. However, this progress in formalizing models and measurement of inner speech (and inner speech judgments) may have come at a cost of ignoring broader elements of the psychology and philosophy of inner speech, such as uses of inner speech (e.g., to problem solve), frequency of inner speech experiences in daily life, and the influence of context (e.g., location, mood) on these perceptions of inner speech.

The focus on daily life experiences of inner speech has been comparatively rarer yet has led to the creation of an assortment of tools that enable one to sensitively and, ideally, reliably measure the experiences of inner speech. Methods that describe inner speech in daily life are subjective/introspective. These methods, therefore, come with their own strengths and weaknesses. While experimental control can be ideal, it can also directly impact the nature and content of the inner speech (e.g., priming, requiring non-spontaneous uses). On the other hand, self-report methods are easy to use, yet are also more prone to inter-individual variability [for more detailed reviews of reliability and validity issues with self-talk measures, see Brinthaupt and Morin (2023) and Van Raalte et al. (2019)]. To summarize: assessing inner speech *in situ* – as it happens – is inherently difficult yet comes with many benefits. Below are several commonly used methods that have evaluated inner speech in daily life.

Methods of sampling behavior in everyday life go by many names, such as experience sampling, ecological momentary assessment, and real-time data capture (Hektner et al., 2007). This type of assessment is defined by three critical features: people are assessed in their natural environments; researchers attempt to measure events or experiences in real-time; and researchers intensively assess people over time, such as several assessments per day across a week (Hektner et al., 2007). Experience sampling is appealing to measure inner speech because inner speech is known to vary by context and thought to not be present all of the time (Hurlburt et al., 2021). Therefore, using intensive methods in a real environment may most sensitively and accurately reflect experiences of inner speech. As with every other method that aims to measure inner speech, there are benefits and drawbacks to experience sampling. A major benefit is the large amount of data collected and the ability to evaluate within-subject and between-subject variability. For example, the collection of seven assessments per day for seven days in 20 participants elicits 980 datapoints and robust statistical power to detect potentially small effects. However, a logical barrier with these designs is a lower rate of compliance and a higher rate of attrition than might be expected from a cross-sectional design, and/or a design confined to a laboratory setting (Silvia and Cotter, 2021). Another benefit is its ecological validity (its ability to reflect information from real life), but a drawback is generally considered to be its lower experimental control (and, therefore, increase in statistical noise). Note, though, that experience sampling can still have some experimental control (e.g., development of a valid assessment protocol at each sample collection), though it is different from the control within a lab environment.

There is no typical or best practice standards about number of times, or duration of sampling, for experience sampling. Results of a recent meta-analysis across research fields demonstrated that, on average, experience sampling studies in neurotypical populations scheduled six assessments per day, lasted for seven days, and obtained a compliance of 79% (Wrzus and Neubauer, 2023). Note that most individuals being sampled were young adults with no concomitant clinical conditions. The authors found that studies with more assessments per day scheduled fewer assessment days but did not find that the number of assessments predicted compliance or dropout rates (Wrzus and Neubauer, 2023). A review evaluating mobile experience sampling in clinical populations found that the median duration of studies was 12 days and the median prompt frequency was five per day across clinical and non-clinical samples (Williams et al., 2021). There was no significant relationship between compliance and prompts per day or items per prompt in clinical samples (Williams et al., 2021).

Experience sampling has been largely used to evaluate the experience of inner speech in young adults, typically undergraduate students. In one study, inner speech was measured six times per day at pseudo-random intervals, for three separate days (Heavey and Hurlburt, 2008). Participants received a notification from a beeper and were asked to describe their inner experience in that moment. Within 24 h, they completed an unstructured interview with the experimenters, detailing their inner experience during the six sampled times (Heavey and Hurlburt, 2008). As previously mentioned, inner speech was found to be a common form of inner experience, with an overall frequency of 26% of sampled instances (with a wide range: 0–75%). In another study by the same group, also with young adults as participants, Hurlburt et al. (2016) combined experience sampling with neuroimaging, with participants reporting if they were experiencing inner speech just before a beep while in an MRI scanner. This study too found an overall frequency of inner speech of 29% across all participants. Authors found that elicited inner speech (rhyme judgments) and spontaneous inner speech (experience sampling) showed differences in activation of Heschl’s gyrus (includes primary auditory cortex) and the left inferior frontal gyrus (activated in language production). Elicited, non-spontaneous inner speech was associated with a significant decrease in activity in Heschl’s gyrus and a significant increase in left inferior frontal gyrus whereas spontaneously occurring inner speech was associated with a significant increase in activity in Heschl’s gyrus but no significant change in left inferior frontal gyrus compared to a baseline. These areas are often part of the injuries which cause aphasia, emphasizing the interest in inner speech in aphasia and the differences in neural patterns of inner speech according to elicitation method.

Questionnaires and surveys are another means of assessing inner speech, and complement other methods by highlighting potentially shared uses of inner speech across a population (Morin and Racy, 2022). Through data obtained with a thought-listing procedure, in which participants wrote about their inner speech over the past six months, Morin and colleagues developed the General Inner Speech Questionnaire (GISQ) (Racy et al., 2020). The General Inner Speech Questionnaire focused on specific themes of when participants talk to themselves (during what activities), what they talk to themselves about (the contents), and why they talk to themselves (the functions) (Racy et al., 2020). In five studies conducted by Morin and colleagues from 2011 to 2019 using thought-listing and the General Inner Speech Questionnaire, they found that the most frequently reported contents and functions of inner speech in neurotypical young adults included problem solving, negative emotions, emotional control, planning, and self-motivation (Morin and Racy, 2022). There are other questionnaires with slightly different aims; for example, the Varieties of Inner Speech Questionnaire-Revised (Alderson-Day et al., 2018) assesses the phenomenology of a person’s inner speech (e.g., whether it is a dialogue or monologue and if it contains all phonetic information) and the functions of inner speech (e.g., self-evaluative, self-regulatory) (Alderson-Day et al., 2018). The Self-Talk Scale (Brinthaupt et al., 2009) focuses on aspects of self-regulation, such as self-criticism, −reinforcement, and -management.

Advantages to questionnaires and surveys are that they can be tailored to specific research questions with theoretical bases, and they provide quantitative results about those specific questions, with some reporting preliminary construct validity and psychometric qualities (Brinthaupt et al., 2009; Racy et al., 2020). A limitation to questionnaires is that they are retrospective and require long-term memory, and as participants recall what happened in the past, their memories may be inaccurate (inflating or underestimating certain experiences of inner speech). Questionnaires may also be less sensitive to subtle changes in context, and they may be affected by the researcher’s preconceptions about the phenomenon of inner speech.

### Aims, hypotheses and rationale for the current study

Most of the research on inner speech in aphasia thus far has been in the context of experimentally controlled, non-spontaneous tasks, such as confrontation naming, word retrieval, and silent rhyme/homophone judgments (for an overview, see Fama and Turkeltaub, 2020). To our knowledge, a single study has used experience sampling methodology to evaluate a cognitive component in adults with acquired, post-stroke aphasia: Sather et al. (2017) used experience sampling to investigate “flow state” (intense concentration often resulting in distorted time passage and loss of self-consciousness) in people with aphasia. No study to date has used experience sampling to investigate inner speech in aphasia. The feasibility of this methodology to sample inner speech in aphasia, and the frequency and themes of inner speech experienced during daily life by adults with aphasia, remain unclear.

The following exploratory, preliminary feasibility study was approved by Indiana University (IRB #10549) and was pre-registered on Open Science Framework.^1^ There were hypotheses detailed in the pre-registration, described in more detail, below. Registered exploratory analyses also included the exploration of the association of inner speech with language impairment awareness (or an impairment in this, “anosognosia”) and with demographic variables, such as age, years post-stroke, and sex.

#### Exploratory research question 1: explore the extent to which individuals with aphasia can reliably engage with descriptive experience sampling and multiple surveys

A compliance of about 80% was expected, given the evidence from prior research (in aphasia: Sather et al., 2017; in adults without brain damage: Wrzus and Neubauer, 2023).

#### Exploratory research question 2: frequency of inner speech in individuals with aphasia

Adults with aphasia have been shown to have varying levels and perceptions of inner speech, measured in a variety of ways though always at the single word level (e.g., rhyme judgments, picture naming) and non-spontaneously. Therefore, it remains unclear how inner speech presents itself spontaneously in daily life in this population. Inner speech in daily life has been shown to be highly heterogeneous in young adults (from 0–75% across different studies) (Brinthaupt et al., 2009; Hurlburt et al., 2021). Therefore, it was difficult to motivate a directional hypothesis, and it is therefore posed as an exploratory question: to what extent do people with chronic aphasia have inner speech in their daily lives?

#### Exploratory research question 3: case analysis

Because of the novel procedures and topic (inner speech), a detailed case-by-case presentation (see Tables 1–4) is also provided to explore potential patterns in the data. Exploratory analyses include an investigation of the relationship between inner speech and selected demographics (age, sex, and years post-stroke), and with a neuropsychological measure, the severity of language awareness impairment (i.e., anosognosia).

**Table 1:** Individual participants with demographic information.

ID	Years post-onset aphasia	Age at testing	Sex	Education (years)	Race	Is Hispanic or Latino?	Bilingual?
2	21.50	50	Female	18	White	No	No
3	0.81	75	Male	22	White	No	No
5	3.98	68	Male	14	White	No	No
6	25.00	45	Female	16	Asian	No	Yes (Hindi, L1; English, aged 5)
7	4.00	52	Male	16	White	No	No
8	10.29	62	Male	18	White	No	Yes (Hebrew L1; English, most of life)
10	2.64	45	Female	18	White	No	No
11	7.38	47	Female	16	White	No	No
14	2.87	62	Female	20	Unknown/not reported	No	No
16	4.43	62	Male	16	White	No	No
19	5.85	63	Female	14	White	No	No
20	1.88	71	Male	14	White	No	No
22	2.58	52	Female	12	Black or African American	No	No
24	6.79	59	Female	18	White	No	No
25	1.67	56	Male	16	White	No	No
29	7.00	67	Female	16	White	No	No
30	10.00	50	Male	20	Asian	No	Yes (English L1; Cantonese for 30+ years)
35	3.00	63	Male	16	More than one race	No	No
46	3.97	38	Female	20	White	No	No
47	16.34	46	Female	17	American Indian/Alaska Native	Yes	Yes (English and Spanish simultaneous)
48	11.00	54	Female	18	White	No	No
63	5.71	58	Female	14	White	No	No
78	7.00	70	Female	18	White	No	Yes (Russian, Czech, Hungarian L1s/simultaneous; English from age 8)
Mean (SD) or percentage Min, max	7.20 years (6.24) 0.81, 25.00	57.2 years (9.78) 37.8, 75.1	60.87% Female	16.8 years (2.39) 12, 22	73.91% White 8.70% Asian 4.35% Black or African American 4.35% More Than One Race 4.35% American Indian/Alaska Native 4.35% not reported	4.35% Hispanic or Latino	21.74% bi- or multilingual

**Table 2:** Individual participants with neuropsychological data.

	Quick aphasia battery									VATA-L		
ID	Overall	Severity	Word comp.	Sentence comp.	Word finding	Grammatical construction	Speech motor programming	Repetition	Reading	Discrepancy*	Participant	Other
2	8.35	Mild	10	3.33	9	9.63	10	8.75	10	−10	15	5
3	8.25	Mild	10	4.58	8.25	9	10	8.75	9.58	1	15	16
5	9.01	Latent/no aphasia	10	6.67	9	9.5	10	8.33	10	8	16	24
6	7.19	Moderate	10	7.08	6.25	3.75	10	6.67	8.75	1.5	17	18.5
7	7.39	Moderate	10	3.33	6.75	8.88	10	6.67	8.33	0	19	19
8	8.7	Mild	10	5.83	7.75	9.5	10	7.92	10	1	8	9
10	9.6	Latent/no aphasia	10	10	9	10	10	10	10	−4	10	6
11	8.85	Mild	10	8.33	8	8.38	10	10	9.58	8	5	13
14	8.08	Mild	7.92	7.92	8.5	8	7.5	8.33	9.58	0	16	16
16	8.8	Mild	8.75	6.67	9	9.38	10	9.58	9.58	−4.5	15.5	11
19	7.9	Mild	10	4.58	9.75	9.75		8.33	9.58	−22	37	15
20	7.88	Mild	10	10	8.5	5.5	5	6.67	7.5	−7	22	15
22	7.47	Moderate	9.58	7.08	5	8.75	7.5	6.67	5.83	−4	24	20
24	8.77	Mild	10	9.58	8	7.5	10	8.33	8.33	0	18	18
25	9.36	Latent/no aphasia	10	8.75	9	9.5	10	10	10	3	1	4
29	9.41	Latent/no aphasia	10	10	10	8	10	10	10	1	4	5
30	9.55	Latent/no aphasia	10	9.17	10	9.88	7.5	9.58	10	−16.5	24.5	8
35	7.66	Mild	10	8.75	7.25	7	10	7.5	0^	−7.5	30.5	23
46	9.76	Latent/no aphasia	10	10	9	10	10	10	10	−4	9	5
47	9.65	Latent/no aphasia	10	10	10	9.88	7.5	9.58	10	7	6	13
48	8.49	Mild	8.33	8.33	4	10	10	9.17	10	5	8	13
63	8.88	Mild	10	8.33	7.5	8.38	10	9.58	10	−1	9	8
78	7.38	Moderate	10	9.58	5.5	6	5	6.25	8.33	−8	21	13
Mean (SD) or percentage Min, max	8.54 (0.81) 7.19, 9.76	30.4% latent 52.2% mild 17.4% moderate	9.76 (0.59) 7.92, 10.0	7.73 (2.17) 3.33, 10.0	8.04 (1.63) 4.00, 10.00	8.53 (1.64) 3.75, 10.0	9.09 (1.64) 5.0, 10.0	8.55 (1.29) 6.25, 10.0	8.91 (2.21) 0, 10.0	−2.30 (7.35) −22.0, 8.0	15.2 (8.80) 1.0, 37.0	12.9 (5.95) 4.0, 24.0

Comp. = Comprehension; VATA-L = Visual Assessment of Anosognosia of Language Impairment. Severity: latent/no aphasia (8.90-10), mild (7.50-8.89), moderate (5-7.49). * = Discrepancy score of a more negative value indicates a higher probability of anosognosia related to language impairment. ^ = Result due to self-disclosed visual perception issues; all text made larger and read aloud to participant (via software on their own phone and also during experiment).

**Table 3:** Individual participants with inner speech data: in the moment inner speech.

ID	Yes (%)	No (%)	Not sure (%)	Compliance (% out of possible 30)
2	84.85	15.15	10	100
3	100	0	0	100
5	78.79	6.06	12.12	96.97
6	72.73	9.09	15.15	96.97
7	100	0	0	100
8	78.79	9.09	9.09	96.97
10	51.51	42.42	6.06	100
11	90.91	9.09	0	100
14	81.82	3.03	0	84.85
16	100	0	0	100
19	57.58	36.36	6.06	100
20	21.21	63.64	0	84.85
22	90.91	3.03	0	93.94
24	69.70	12.12	3.03	84.85
25	93.94	3.03	0	96.97
29	90.91	9.09	0	100
30	36.36	21.21	0	57.58
35	93.94	6.06	0	100
46	96.94	0	0	93.94
47	100	0	0	100
48	81.82	9.09	0	90.91
63	78.79	21.21	0	96.97
78	54.55	30.30	15.15	100
**Mean (SD)**	78.26 (21.74)	13.33 (15.83)	3.04 (5.31)	94.64 (10.33)
**Range (min, max)**	23.33, 100	0, 63.33	0, 16.67	57.58, 100

“In the moment” instances constituted 30 possible prompts per participant, resulting in a total of 690 possible responses across the study (30 prompts x 23 participants).

**Table 4:** Individual participants with inner speech data: General Inner Speech Questionnaire.

ID	Compliance (% out of 3 completed)	Total “content” (I talk to myself about)			Total “function” (I talk to myself in order to)			Total “activities” (I talk to myself when)			Total items		
*Week*		*1*	*2*	*3*	*1*	*2*	*3*	*1*	*2*	*3*	*1*	*2*	*3*
*Maximum allowed*		*16*			*11*			*5*			*32*		
2	100	14	12	10	7	6	5	5	1	3	26	19	18
3	100	13	13	16	10	9	10	5	5	5	28	27	31
5	100	4	3	3	3	2	2	2	1	1	9	6	6
6	100	11	8	7	5	5	7	3	2	4	19	15	18
7	100	8	10	13	7	9	7	5	5	5	20	24	25
8	100	9	7	8	7	2	4	4	2	2	20	11	14
10	100	15	11	14	9	7	7	2	2	4	26	20	25
11	100	16	15	16	10	10	10	5	5	5	31	30	31
14	100	10	12	10	7	6	8	3	3	5	20	21	23
16	100	10	9	7	7	9	9	4	4	4	21	22	20
19	100	3	13	6	6	8	6	2	3	2	11	24	14
20	100	12	4	4	9	1	2	5	1	1	26	6	7
22	100	10	5	12	7	6	5	3	2	2	20	13	19
24	100	11	11	14	8	8	9	3	3	4	22	22	27
25	100	13	10	14	9	4	9	3	2	3	25	16	26
29	100	5	5	5	7	7	7	2	2	2	14	14	14
30	33.33	10	NA	NA	9	NA	NA	4	NA	NA	23	NA	NA
35	100	3	14	2	3	7	3	0	3	0	6	24	5
46	100	11	9	11	8	6	9	3	4	4	22	19	24
47	100	16	16	16	10	10	11	4	4	4	30	30	31
48	66.66	14	15	NA	9	10	NA	3	3	NA	26	28	NA
63	100	5	7	5	5	7	7	4	2	4	14	16	16
78	100	12	7	13	8	9	9	3	5	3	23	21	25
**Mean (SD)**	95.65 (15.26)	10.22 (3.97)	9.82 (3.76)	9.81 (4.56)	7.39 (1.99)	6.73 (2.64)	6.95 (2.64)	3.35 (1.27)	2.91 (1.34)	3.19 (1.47)	20.96 |(6.51)	19.45 (6.84)	19.95 (7.97)
**Range (min, max)**	33.33, 100	3, 16	3, 16	2, 16	3, 10	1, 10	2, 11	0, 5	1, 5	0, 5	6, 31	6, 30	5, 31

The General Inner Speech Questionnaire (adapted) was filled out on three different occasions by each participant, thus constituting a total of 69 possible surveys across the study (3 surveys × 23 participants).

#### Hypothesis 1: content, functions and activities of inner speech

We hypothesized that people with aphasia likely have some shared contents of inner speech with each other and with young adults, but they may have unique themes or have certain themes that are more prominent because of their age and/or brain injury. As an exploratory part of this hypothesis, we expected that inner speech would not be consistent over three weeks, as it is a heterogeneous experience in young neurotypical adults, known to be impacted by context (Alderson-Day and Fernyhough, 2015). To our knowledge, no study (in a clinical or non-clinical sample) has systematically evaluated the consistency of inner speech experience in this way, making this a highly exploratory question.

#### Hypothesis 2: relationship of inner speech with aphasia severity

Prior research is divided on the relationship between aphasia severity and inner speech, and the method of measuring inner speech may affect whether it relates to aphasia severity (Alexander et al., 2023). In Alexander et al. (2023), self-reported inner speech (the subjective ratings of inner speech in general and successful inner speech during naming) did not relate to aphasia severity. Experience sampling has not been used to study inner speech in aphasia before, but it is similar in that it requires self-report, so no relationship between aphasia severity and inner speech was expected.

## Method

### Participants

Participants were recruited over a period of one year. They were proficient English speakers (defined by self-report; speaking another language did not exclude), aged 18–80 years old, and had aphasia due to a stroke occurring at least six months prior. To ensure that auditory comprehension abilities were strong enough to participate in the study, participants were required to score 30 or greater (out of 48) on the auditory sentence comprehension subtest of a short, standardized aphasia battery [Quick Aphasia Battery (QAB); Wilson et al., 2018]. If a participant scored lower than that, study personnel examined their comprehension of verbal instructions, written communication, and practice with procedures to ensure adequate comprehension to include them (this occurred for two participants, who were ultimately included). One exclusion parameter involved the presence of disorders that may make their speech unintelligible, such as severe apraxia of speech or dysarthria. Speech motor impairments were rated using the speech motor programming section of the Quick Aphasia Battery (see Table 2 for Speech Motor Programming scores). No participants were excluded due to a severe motor speech impairment. The final exclusion parameter involved the self-report of any other neurological disorder (e.g., Alzheimer’s disease); no exclusions were made based on this.

Verbal consent was acquired for 26 adults with aphasia. Two adults with aphasia dropped out after the introductory session/a few experience sampling responses due to busyness (did not have time to complete all study components and proactively dropped out because of this). It was realized at the end of the study that one adult with aphasia had a concomitant stroke and traumatic brain injury, and their data are excluded from these results. Detailed demographic and neuropsychological data for the final sample of 23 is shown in Tables 1, 2.

### Procedure and materials

**Figure 1:**
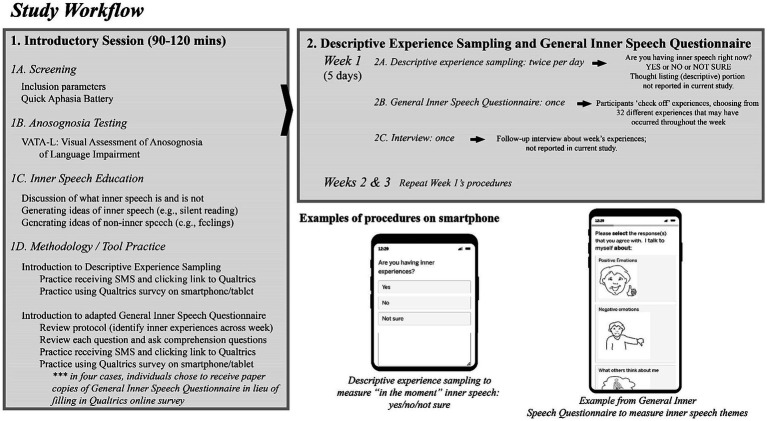
Workflow of study procedure.

Participants took part in a three week long mixed methods study, containing four primary components: (1) Introduction to inner speech and the study procedures, (2) Experience sampling, (3) General Inner Speech Questionnaire (Racy et al., 2020), and (4) Interviews (Figure 1). All procedures were conducted remotely and virtually using secure Microsoft Teams platform (Introduction; Interviews), Qualtrics (General Inner Speech Questionnaire, Descriptive Experience Sampling), and Apple iPhone Automations (SMS notification to engage with Qualtrics).

The mixed methods design was an embedded one, with a primary emphasis on quantitative data, which are reported here (Creswell, 2007). The qualitative data (interview and thought listing) was meant to enhance and elaborate on the quantitative at a later date. The qualitative results are not reported in this paper and are currently being analyzed.

### Data collection

#### Introductory session

There were several purposes to the Introductory session: (1) screening for inclusion and collecting more information about aphasia characteristics, (2) characterizing insight into language impairment, (3) introducing the participant to “inner speech” and its definitions, and (4) practicing the technology/methods used in the study. The motivation for the multiple purposes of the Introductory session was due to both Morin and Hurlburt’s inner speech research emphasizing the need to create a shared understanding of what inner speech is and is not (Hurlburt and Akhter, 2006; Heavey et al., 2019; Racy et al., 2020; Hurlburt et al., 2021; Brinthaupt and Morin, 2023); for the purposes of more comprehensively documenting each person’s demographic and neuropsychological characteristics; and because of our prior research using virtual testing in aphasia emphasized that troubleshooting technology and methodology together leads to better outcomes and low attrition (Doub et al., 2021; Stark et al., 2023). Note that Hurlburt’s *descriptive* experience sampling methodology was not employed in its entirety in this study, as that methodology is highly iterative and involves interviews after each day, with many instances of discussing what inner speech is and is not after each sampling period.

The typical Introductory session lasted between 90–120 min and occurred entirely on Microsoft Teams. Several breaks were given to mitigate fatigue.

##### Screening for inclusion and characterization of aphasia

Prior to enrolling in the study, participants were emailed a link to a REDCap survey, where they self-reported demographic information about inclusion and exclusion parameters (Harris et al., 2009, 2019). If found to be potentially eligible, they were invited to the virtual Introductory session. During the Introductory session, the screening for inclusion involved verifying that auditory comprehension was adequately preserved for the purposes of participation, as described under Participants. The Quick Aphasia Battery was also used to further characterize each participant’s aphasia (Table 2).

##### Characterizing insight into language impairment

If eligible to participate in the study, participants were administered the VATA-L: Visual-Analogue Test assessing Anosognosia for Language Impairment (Cocchini et al., 2010) to assess insight into language deficits. Assessing anosognosia (lack of insight into a particular disorder or disease) in aphasia has traditionally been very difficult, with few validated assessments available. The VATA-L is one such way of evaluating anosognosia specific to language impairment, and has the participant, as well as a familiar peer (e.g., significant other, speech therapist), use a visual analogue scale to identify the severity (0 = no difficulty; 1 = some difficulty; 2 = difficult; 3 = very difficult) of communication difficulty during common language production and comprehension situations (e.g., writing sentences, getting the sounds of a word, picking up mistakes in your speech, reading notices, understanding people). The VATA-L also contains several “check” items to ensure that the anosognosia does not extend beyond language (e.g., VATA-L asks about awareness of difficulty waving, hearing a fire engine with sirens on, and jumping over a truck/lorry).

##### Introduction to inner speech

It was very important for the fidelity of the study to ensure that all participants understood what “inner speech experience” was and was not, a clarification that has been made clear by other researchers using similar methods to study inner speech (Racy et al., 2020; Hurlburt et al., 2021). Therefore, following screening and further characterization of aphasia and insight into language impairment, the Introductory session involved a detailed discussion (also using visual aids) between the participant and author JA about what inner speech is and is not (Table 5). Table 5’s exemplar inner speech examples were generated between authors JA, BCS, and inner speech expert, Alain Morin, prior to the study’s initiation. After discussing the exemplars in the table, JA had participants generate other examples of what inner speech is and is not to ensure their understanding. Some representative examples from participants that were considered inner speech included: reading to oneself, praying, planning out a conversation and hearing those words, and singing to oneself. Some representative examples from participants that were correctly not considered inner speech included: feeling sad, and “seeing” an item in their head but not having the word for it (like an “Idea Without Word” concept, as described in Fama et al., 2019b; Fama and Turkeltaub, 2020).

**Table 5:** Inner speech definitions.

Inner speech is:	Inner speech is NOT:
Verbal (thinking)	Non-verbal thinking
A commentary	Silent thinking
In words	Sensations
Self-talk	Images
Inner speaking	Pure emotions
Talking to yourself	Wordless thinking
In your own voice	
Words or sentences	
Mostly silent	
Monologue or dialogue	
Meaningful or nonsensical	
More or less frequent	
Spontaneous or voluntary	

This table was generated as a result of discussions with Alain Morin, PhD, and was used in the Introductory session of the study.

##### Practicing methodology for study

A core part of the study was adherence to procedures and the use of (potentially) new technology. Therefore, the end of each Introductory session focused on practicing the methodology with author JA.

Participants practiced receiving the SMS notification to start the experience sampling procedure (this procedure is described in more detail, below). Each participant was sent an SMS by author JA, and they were tasked with opening the link in the text message and going through the protocol on Qualtrics (see Figure 1 workflow). Author JA verified that the Qualtrics response had been received and probed about the participant’s experience, ensuring they understood how to use the technology.

Finally, JA and the participant practiced answering the General Inner Speech Questionnaire, reinforcing the need to accurately represent the participant’s experience of inner speech, including that participants should leave items blank if they did not experience them. If participants indicated that they preferred a paper copy rather than doing either the experience sampling or the General Inner Speech Questionnaire survey via Qualtrics, a paper packet was shipped to their home with return postage included. Four participants opted for the paper packets. The paper packets are located on the OSF repository for this study.^2^

#### Experience sampling methodology

The goal of the current study was to acquire 30 experiences per participant, in line with the best practices suggested by Hurlburt and Akhter (2006). Experience sampling methodology, broadly, aims to understand behavior as it occurs in daily life. Each experience sampling occurrence prompted each participant first about whether or not they were experiencing inner speech, and then asked to elaborate on it via a thought listing methodology (discussed below). For the purposes of this quantitative study, only the experience sampling component (whether or not they were experiencing inner speech) is reported.

In the current study, the experience sampling procedure took place over three weeks, five days per week, twice per day. The time of the sampling was pseudo-random per day (Hektner et al., 2007), where participants received probes between 9:00–10:00 AM and 4:00–5:00 PM. The times differed only if a participant indicated that they were not usually awake at those times (i.e., one participant requested a later hour for the first prompt because he is a late riser). This pseudo-random design was chosen to limit poorly timed disruptions to participants’ daily activities and increase their ability to respond to prompts immediately, thereby maximizing construct validity. The choice of using two probes per day was made as a means of compromising on feasibility and compliance alongside collecting enough total data-points per participant.

Participants were sent a prompt via SMS (text message), which included a link to the short survey in Qualtrics. The Qualtrics link began with having participants answer a simple question: were you having inner speech when the alarm/beep was received? The response options were “yes,” “no,” and “not sure” (see Figure 1). This data is referred to as “in the moment” inner speech. Or, if the participant had opted for the paper version, the SMS message indicated that they should fill out their paper copy.

There was also a section of the Qualtrics survey where participants were prompted to make notes about their experience, if they answered ‘yes’ to the “are you experiencing inner speech” prompt. This constituted the descriptive portion of the study. This ‘thought listing’ could be done via entering text from the keyboard, recording their own voice (via the Phonic component of Qualtrics), or by uploading a picture of something they had written or drawn. An additional component (an interview) was conducted three times throughout the three-week design, in which inner speech experiences were further probed. During these interviews, author JA also resolved any questions or issues that arose during the week related to the study. The thought listing and interviews constituted the “explore” qualitative component of this mixed method project, and per the recommendation of Creswell (2007), these qualitative data will be presented in more depth in a following publication. They are presently being analyzed and are not included in this quantitative study.

#### General inner speech questionnaire

At the end of each of the three weeks, an additional SMS message was sent to individuals which contained the link to the adapted General Inner Speech Questionnaire. Or, if the participant had opted for the paper version, the SMS message indicated that they should fill out their paper copy. Following an “in the moment” question (are you experiencing inner speech right now?), the participants were then asked to think about their inner speech throughout the week. The requirement of reflection over the course of the week was stated in the questionnaire itself and also discussed whilst practicing the General Inner Speech Questionnaire during the Introductory session. The General Inner Speech Questionnaire asks about the presence or absence of inner speech in three sections: “I talk to myself about” (contents), “I talk to myself in order to” (function), and “I talk to myself when” (activities). The original General Inner Speech Questionnaire contains 57 total items (Racy et al., 2020). For the purposes of this study, an adapted, shortened version of the General Inner Speech Questionnaire was developed between the author team and Alain Morin, PhD, senior developer of the General Inner Speech Questionnaire. First, items that were infrequently chosen by young adults in past administrations of the General Inner Speech Questionnaire were removed, such as thinking about education or studying. Then, items that may be inapplicable to, or insensitive to include, were pre-emptively removed, such as ‘when working’ (in case of forced retirement). For all removals, see Supplementary Table S1. The wording of three items from the original survey was modified: “about how I am perceived by others” was reworded more simply to “what others think about me,” “using language” was clarified to be “when reading,” and “leisure” was broadened to “what I want to do.” This resulted in 32 items on the adapted version. Finally, black and white drawings (free clipart) accompanied each item in the adapted version to clarify their meaning. There were between five and six drawings/choices on each page of the paper packet.

After each of the three sections—“I talk to myself about” (contents), “I talk to myself in order to” (function), and “I talk to myself when” (activities)—there was a 1–5 Likert-scale that probed the participant’s confidence about how accurately they recalled their inner speech for that section. Above the 1 marker were the words “not confident” and a black/white clipart of a person with thumb pointing down, and above the 5 marker was the word “very” and a black/white clipart of a person with thumb pointing up. In prior work in neurotypical adults, most tended to be fairly confident to very confident (scoring between 3 and 5) (Racy et al., 2020), and thus this was expected to be reflected in the current exploratory study using the adapted General Inner Speech Questionnaire in aphasia.

**Figure 2:**
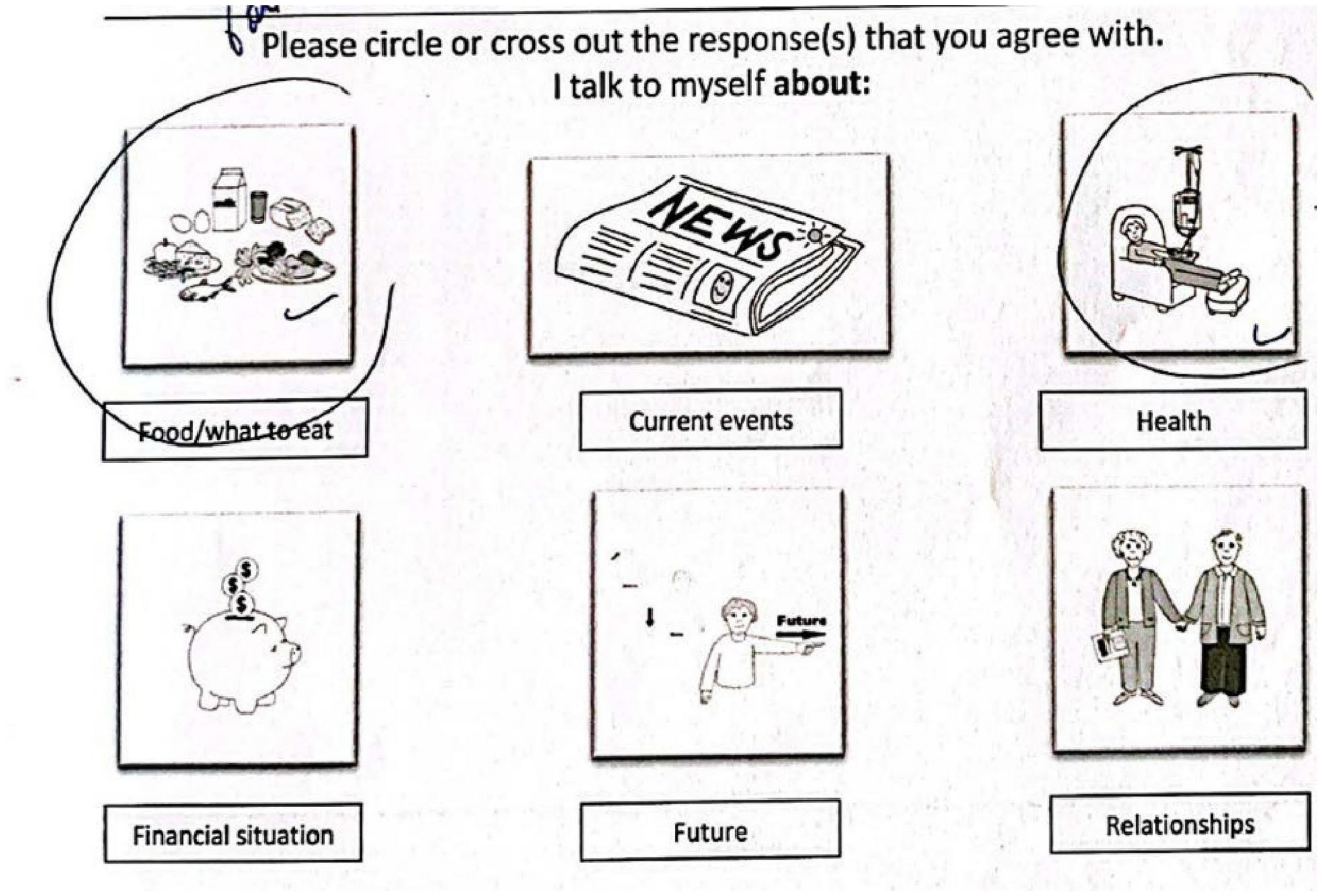
Real example of response using paper packet adapted General Inner Speech Questionnaire.

For an example of one page of the adapted General Inner Speech Questionnaire filled out on the paper form, see Figure 2. For an example of how the General Inner Speech Questionnaire looked on a web browser, tablet and mobile phone as displayed through Qualtrics, see Figure 1. The adapted General Inner Speech Questionnaire used in this study (including pictures) is located in Supplementary File S1. For ease of access, all the materials used in the current study are available on the Open Science Framework (see text footnote 2).

### Data analysis

All analyses were computed using R 4.3.2 and RStudio 2023.12.0 Build 369. Outliers are identified in each section (using boxplots generated in R) but were kept in analyses because of the preliminary nature of the data and overall desire to characterize the whole sample.

#### Exploratory question 1: procedure compliance

To evaluate the extent to which participants could complete the rigorous procedures, the number of responses received from participants was divided by the total number of notifications sent to each participant to complete the experience sampling (30 times) and the number of General Inner Speech Questionnaire surveys completed (out of three chances). This provided a compliance percentage for each portion of the study.

#### Exploratory question 2: frequency of inner speech

The experience sampling “are you having inner speech right now” data (including the three times this question was also asked on the General Inner Speech Questionnaire) were summed. For analyses, the proportion of “yes” responses for the “in the moment” data were used. There were very few “not sure” answers throughout (~3%). Descriptive analyses were used to quantify the frequency of response types.

#### Exploratory question 3: case analysis

Exploratory analyses include an investigation of the relationship between inner speech and selected demographics (age, sex, and years post-stroke), and with a neuropsychological measure, the severity of language awareness impairment (i.e., anosognosia). Appropriate correlation tests (Spearman’s or point-biserial) were conducted to evaluate these relationships, and power (β) is also included to aid interpretation because of the known small sample size. Power was calculated using the *pwr* package in R 4.3.2. Boxplots of the “in the moment” data were used to reveal outliers, leading to in-depth analysis of their clinical presentation based on subtests of the QAB and the discrepancy score on the VATA-L.

#### Hypothesis 1: content, functions, and activities of inner speech use

A pre-registered hypothesis revolved around identifying patterns in the General Inner Speech Questionnaire themes across the participants. Descriptive statistics were used to quantify the number of themes within the three categories (content, function, and activities) summed across the three weeks to identify patterns of inner speech experience. This was done for the entire sample (although two individuals did not complete all surveys, see Compliance in Results). To ascertain what percentage of the sample selected a theme, the total number of individuals selecting the theme was summed and then this was divided by the total number of participants. The average confidence ratings for each section of the General Inner Speech Questionnaire overall are also reported. This hypothesis was a null one: that there would be no difference in number of inner speech themes/items between sample with aphasia and prior data collected from young adults. As such, Fisher’s null hypothesis testing protocol was followed.

Despite the research team’s best efforts, it was realized that a question on the General Inner Speech Questionnaire which was on the Qualtrics version was not on the packet version (“Listen to my own voice,” from the functions section). Therefore, the four participants who used the packet do not have data for that question, and results should be interpreted as such. In the written packet version, one item was included twice (“Replay past conversations”). If they selected one or both instances, this was counted as being experienced.

The consistency of inner speech themes across the weeks was evaluated using Friedman tests for total themes of inner speech experienced, and then the number of themes selected within each inner speech category: content (“I talk to myself about”), function (“I talk to myself in order to”) and abilities (“I talk to myself when”).

#### Hypothesis 2: relationship of inner speech to aphasia severity

A pre-registered hypothesis evaluated the relationship of inner speech with aphasia severity. The “in the moment” inner speech data (proportion of “yes” responses) was correlated with aphasia severity (total Quick Aphasia Battery score). The total number of inner speech themes derived from the General Inner Speech Questionnaire (summed across three administrations) was correlated to aphasia severity for the *n* = 21 with all three administrations of the survey. Given this is a null hypothesis, the Fisher null hypothesis testing paradigm was employed, following the classic procedure of stating significance level (alpha = 0.05), calculating test statistic and *p*-value, and then deciding to reject or fail to reject the hypothesis. Correlations were the main statistical test, and power (β) is also included to aid interpretation because of the known small sample size.

Given that prior research has found relationships with overt speech (more inner speech tends to associate with better overt speech), and with phonological processing, exploratory correlations were run between the inner speech variables (proportion of “yes” in the moment responses; total General Inner Speech Questionnaire themes) and the QAB Word Finding and QAB Repetition subtests. Correlations were the main statistical test, and power (β) is also included to aid interpretation because of the known small sample size.

## Results

### Exploratory question 1: procedure compliance

For the 23 individuals included in the study, there was 94.64% (SD = 10.33) compliance for the “in the moment” inner speech assessments and 95.65% compliance on the General Inner Speech Questionnaire. There were three outliers identified for the experience sampling portion: one with a compliance of 87%, one with 83%, and one with 57%. There were two outliers identified for the General Inner Speech Questionnaire administrations: one with 67% (completed two of three) and one with 33% (completed one of three). The two dropouts noted in the Methods are considered attrition, and therefore, there was a total participant attrition rate of 7.69% (26 enrolled, two dropping out prior to study completion). The excluded individual (due to late discovery of other non-eligible brain injury) completed all study procedures but is not further reported on and not considered part of attrition or compliance.

There were three participants who answered the General Inner Speech Questionnaire more than three times (although sometimes incomplete), and only the three times that corresponded with when they were sent the notification (weekly on day 5 in the evening) were counted.

As this was an exploratory study for the procedures being implemented in this specific population, the research team wanted to evaluate the type of technology that was used by participants to complete the procedures. No rules were implemented on what kind of technology could be used. In the study, 13.04% used a tablet, 78.26% used a mobile phone, and 4.35% used a computer. 17.39% opted to use the paper packet at least once over the course of the procedure.

### Exploratory question 2: frequency of inner speech

**Figure 3:**
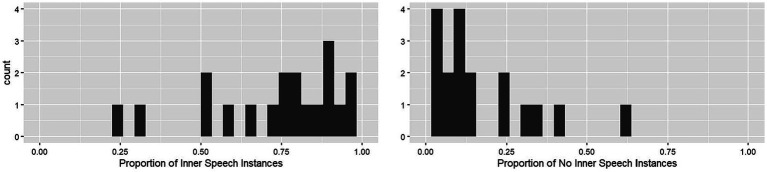
Frequency of “in the moment” inner speech occurrences across the experience sampling paradigm. The y-axis represents a count of participants experiencing inner speech (x-axis computed as a proportion of responses out of 30), for the presence of inner speech (left) and the non-presence of inner speech (right).

Participants responded to the question “are you having inner speech?” on 30 different occasions. Most of the time (>78%), they reported “yes” to having inner speech in that moment (Table 3; Figure 3). As suspected, there was wide variability in the experiences of inner speech across the study (i.e., 23.23% “yes” answers across study for one participant vs. 100% “yes” answers across study for several participants). There were two outliers identified for the “yes” responses, with 30 and 23%. There was one outlier identified for the ‘no’ responses, with 63% (this participant was also the participant with 23% yes responses). There were three outliers identified for the “not sure” responses, with 13, 13, and 17%. The subject with 17% not sure was also an outlier for the “yes” responses (with 53% yes).

For the responses recorded on the Qualtrics survey (but not for the paper packets), exact timestamps of responses were extracted. Based on anecdotal reports of people with aphasia experiencing exhaustion and corresponding language difficulties later in the day, the proportion of “yes” responses was compared between the morning and late afternoon/evening sampling times. All responses recorded before noon were considered morning, while responses recorded after noon were considered evening, regardless of when the participants were scheduled to receive a prompt. For this subgroup who answered via the Qualtrics survey, the proportion of “yes” responses in the morning was nearly equivalent to the evening (83.7% vs. 82.0%, respectively).

### Exploratory question 3: case analysis

Given the novelty of this study, detailed participant information is available in Tables 1–4, enabling a careful evaluation of each participant’s demographic, neuropsychological, and inner speech variables.

#### Demographic relationships with inner speech

An exploratory Spearman’s correlation did not identify a significant relationship between age and proportion of “yes” “in the moment” responses (r_s_ = −0.10, *p* = 0.65, β = 0.07), or between total types of inner speech experiences across the week during the experiment (i.e., general inner speech questionnaire checked categories summed across the study) (r_s_ = −0.42, *p* = 0.06, *n* = 21, β = 0.49). There were no outliers identified for age. Similarly, an exploratory Spearman’s correlation did not identify a significant relationship between years post-stroke and inner speech variables (“in the moment” inner speech proportion of “yes” responses, r_s_ = −0.16, *p* = 0.46, β = 0.07; total types of inner speech experiences, r_s_ = 0.04, *p* = 0.86, *n* = 21, β = 0.05). Two subjects were considered outliers, having experienced a stroke many years prior to the study (21.5 years and 25 years). Finally, an exploratory point-biserial correlation did not identify a significant relationship between sex and inner speech variables (“in the moment” inner speech proportion of “yes” responses, r_pb_ = −0.02, *p* = 0.93, β = 0.05; total types of inner speech experiences, r_pb_ = −0.29, *p* = 0.20, *n* = 21, β = 0.25).

#### Language impairment awareness relationship with inner speech

Generally, the sample showed great variation in VATA-L discrepancy scores, ranging from −22 (where the participant’s estimate of their abilities far exceeded the peer’s estimate of their abilities, reflective of anosognosia) to 8 (where the peer’s estimate of the participant’s abilities slightly exceeded the participant’s own estimate) (*M* = −2.30 ± 7.35). A more negative discrepancy is indicative of anosognosia, or impaired insight into one’s own language impairment, with one prior study suggesting that a score of lower than 13.1 (in the negative direction, i.e., a more negative score than −13.1) is reflective of anosognosia in aphasia. In the current sample, there were two participants who met that criterion for anosognosia (see Table 2). No discrepancy, or a positive discrepancy, suggests self-awareness or insight into one’s own language impairment. A very high positive value (which we did not observe in this study) would suggest heightened awareness/insight into one’s own language impairment.

Spearman correlation did not identify a significant relationship between “in the moment” inner speech (proportion of “yes” responses) and language impairment insight with inner speech (discrepancy score from VATA-L) (r_s_ = 0.33, *p* = 0.13, β = 0.35), suggesting that those who may have anosognosia (receiving a negative discrepancy score, thus rating themselves higher than the ‘other’ person rated them) are not less likely to say that they have “in the moment” inner speech across the descriptive experience sampling timepoints. The total types (i.e., number) of inner speech themes derived from the General Inner Speech Questionnaire (summed across three administrations) was not significantly related to anosognosia (r_s_ = 0.24, *p* = 0.31, *n* = 21, β = 0.18). Spearman’s correlation showed that aphasia severity (r_s_ = 0.25, *p* = 0.25, β = 0.21) did not demonstrate a significant relationship with language impairment insight, and therefore we did not control for either variable when computing analyses between these variables and the inner speech variables. Note that there were two outliers identified for their VATA Discrepancy scores, both scoring very low (lacking insight), with scores of −16.5 and − 22.

#### Participants with less frequent inner speech

When examining the frequency of inner speech (proportion of “yes” responses for in the moment inner speech) in specific cases, two outliers were identified – Participant 20 and Participant 30. To reflect an accurate characterization of the sample (including heterogeneity) and because of the preliminary nature of the study, these participants are described in a case-wise fashion here. As shown in Table 3, they reported having inner speech less often than the other participants (participant 20 at 21% and Participant 30 at 36%, compared to the group average of 78%). Upon exploring the clinical profiles of these two participants (Table 2), it was clear that Participant 20 had a more severe motor speech impairment than most (a 5 out of 10 on the Speech Motor Programming subtest of the QAB). Interestingly, one other participant demonstrated the same score (5 out of 10) for motor speech impairment and also responded with “yes” less often than the group as a whole (participant 78, who responded with “yes” in 55% of instances per Table 3). Participant 78 was not an outlier, however. Participant 30 had very mild aphasia (latent/no aphasia by the QAB), and a 7.5 out of 10 on Speech Motor Programming, showing some mild motor programming impairments. Participant 30 was highly educated, worked in a demanding job during the course of the study, and was the participant with the lowest compliance in responding to prompts in this study (58% compliance on the “in the moment” prompts compared to the group average of 95%). Participant 30 also had a larger negative discrepancy in VATA-L scores (−16), indicating greater likelihood of anosognosia.

### Hypothesis 1: content, functions, and activities of inner speech use

**Figure 4:**
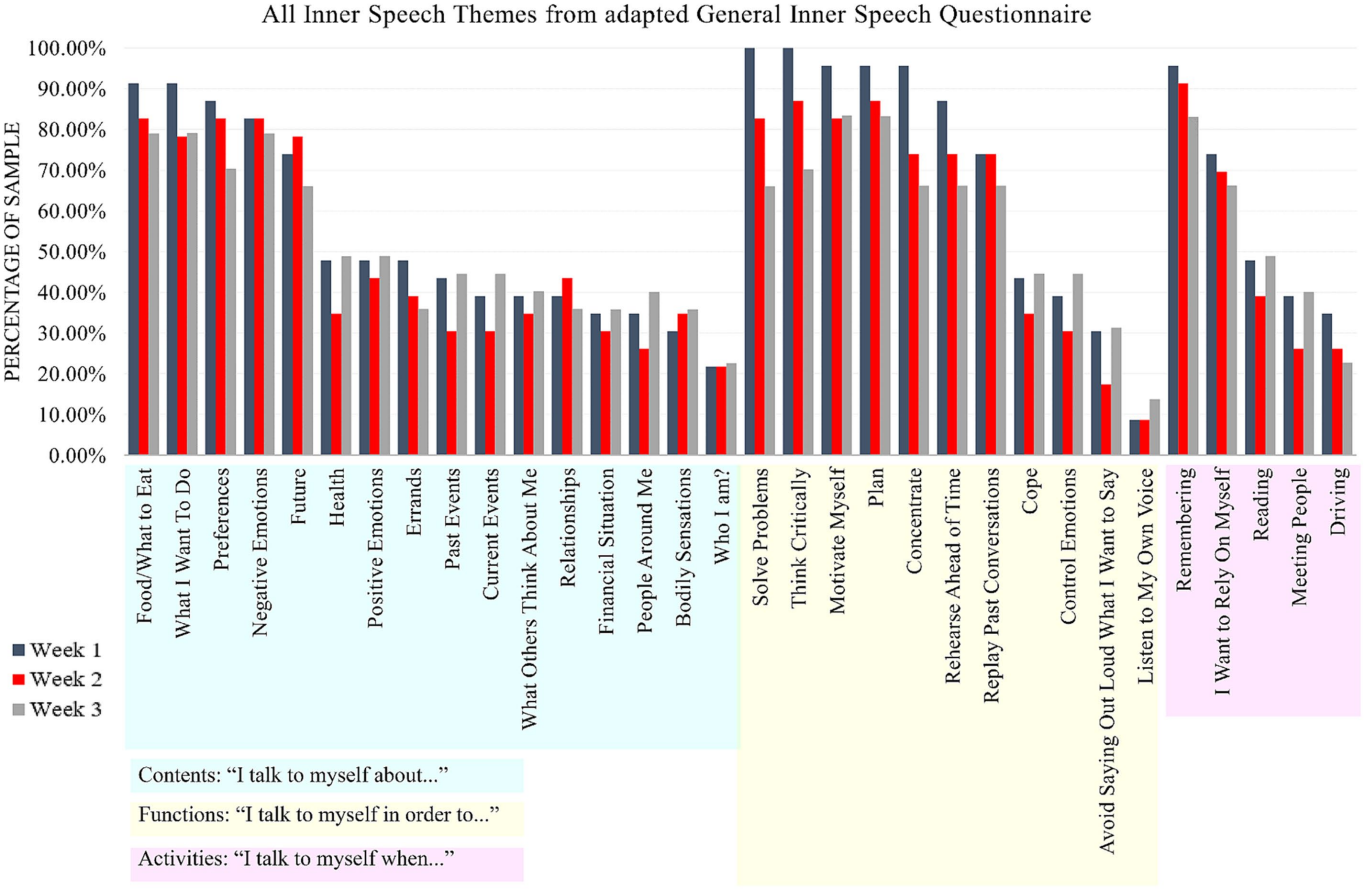
Prevalence of themes of inner speech from the adapted General Inner Speech Questionnaire. There were three administrations of this survey (Weeks 1, 2, 3). The categories of inner speech are highlighted in light blue (“I talk to myself when”), light yellow (“I talk to myself in order to”), and light purple (“I talk to myself when”), to highlight how the themes of inner speech fit into these categories.

The extent to which participants used inner speech to talk about similar things (*content* – “I talk to myself about”), for similar reasons (*functions* – “I talk to myself in order to”) and during similar situations (*activities* – “I talk to myself when”) was characterized by analyzing the percentage of participants who selected a theme across the administrations. For the purposes of characterizing patterns in the data, all participant data (*n* = 23) was used. To streamline Results, a broad overview is presented here and readers are referred to Table 4 and Figure 4 for detailed results.

Across all survey administrations, the highest percentage of participants used inner speech “when remembering”, which was followed closely by using inner speech “to plan”, to “motivate myself”, to “think critically”, and about “food/what to eat”. Other themes that were endorsed, on average, by more than 70% of the participants included using inner speech “to solve problems”, about “what I want to do”, about “negative emotions”, about preferences, in order “to concentrate” and in order “to rehearse ahead of time.” Generally, themes from the General Inner Speech Questionnaire “I talk to myself in order to” section were the most commonly experienced by participants in this study. As hypothesized, patterns across the group were identified alongside variability, which is evidenced in Figure 4. No outliers were identified when evaluating the total number of General Inner Speech Questionnaire themes identified over the course of the three weeks. When evaluating the total number of themes for each theme area (content, functions, activities) across the three weeks (in the *N* = 21 with 100% compliance), no outliers were identified for either “content” or “activities” themes. One outlier was identified for “functions” theme, who identified only seven inner speech functions across the three weeks (compared to a group mean of 20.76).

Participants’ confidence ratings in their answers were very high overall (across all theme areas, max score of 5 [high confidence], Week 1: *M* = 4.54 ± 0.31, Week 2: *M* = 4.35 ± 0.34, Week 3: *M* = 4.00 ± 0.57, Average: *M* = 4.14 ± 0.31), reflecting what had been previously found in neurotypical young adults (Racy et al., 2020) and giving investigators confidence in interpreting the findings as being reflective of participants’ inner experiences. No outliers were identified for confidence across any of the themes.

The similarity of total inner speech experiences across the three weeks was evaluated using a Friedman test, finding that there was not a significant difference in the total number of inner speech themes selected across each of the three weeks in the sample (X^2^_r_ = 2.98, *p* = 0.23, df = 2, *n* = 21). For week 1, *M* = 20.62 themes or 64.43% of all possible themes were chosen; for week 2, *M* = 19.05 themes or 59.52% of all possible themes were chosen; and for week 3, *M* = 19.95 themes or 62.35% of all possible themes were chosen. Three further Friedman tests confirmed that this was likewise the case within each category of inner speech experiences: content (“I talk to myself about,” X^2^_r_ = 2.57, *p* = 0.28, df = 2, *n* = 21), functions (“I talk to myself in order to,” X^2^_r_ = 2.54, *p* = 0.28, df = 2, *n* = 21), and abilities (“I talk to myself when,” X^2^_r_ = 1.12, *p* = 0.57, df = 2, *n* = 21). Given no statistical test was significant, multiple comparison correction was not implemented.

### Hypothesis 2: relationship of inner speech to aphasia severity

The hypothesis was that there would not be a relationship between inner speech and aphasia severity. The statistical tests failed to reject the null hypothesis. There was no significant relationship between the proportion of saying ‘yes’ during “in the moment” inner speech and aphasia severity (r_s_ = 0.11, *p* = 0.63, β = 0.08). There was no significant relationship between total types of inner speech themes selected from the General Inner Speech Questionnaire and aphasia severity (r_s_ = 0.19, *p* = 0.41, *n* = 21, β = 0.13).

Given that prior research has found relationships with overt speech (more inner speech tends to associate with better overt speech), an exploratory correlation was run between the QAB Word Finding subtest, the proportion of saying “yes” during “in the moment” inner speech (r_s_ = 0.03, *p* = 0.90, β = 0.05), and the total types of inner speech themes selected from the General Inner Speech Questionnaire (r_s_ = 0.02, *p* = 0.94, *n* = 21, β = 0.05). Prior research has also broadly implicated phonological output processes as being related to inner speech, and therefore exploratory correlations were conducted between the QAB Repetition subtest, the proportion of saying ‘yes’ during “in the moment” inner speech (r_s_ = 0.24, *p* = 0.27, β = 0.20), and the total types of inner speech themes selected from the General Inner Speech Questionnaire (r_s_ = 0.32, *p* = 0.16, *n* = 21, β = 0.30).

## Discussion

This is a preliminary study reporting on a relatively small and novel sample of the experience of daily life inner speech by persons with chronic, post-stroke aphasia.

### Exploratory question 1: procedure compliance

A goal of this study was to examine the feasibility of conducting a study including naturalistic and introspective sampling procedures in adults with chronic aphasia. Feasibility was defined as demonstrating high compliance with study procedures. The participants in this study demonstrated high compliance for both experience sampling and completion of the longer, once-weekly survey, with only one participant demonstrating a compliance of <83%. There were a few participants (*n* = 3) who completed the General Inner Speech Questionnaire more times than they were asked, which we handled by only accepting the responses from the time points at which the notifications were sent, but this could be seen as a limitation as these few participants did not strictly adhere to the instructions. Future work in this area should ideally limit the number of times an IP address (or similar) can fill out the surveys, therefore mitigating this concern.

Only one study, to our knowledge, has leveraged experience sampling methodology in a study sample of participants with aphasia (to study flow states; Sather et al., 2017). The individuals from the Sather et al. (2017) study all had mild, chronic aphasia. Overall, the Sather sample appears like the sample leveraged in the current study, which likewise involved individuals with chronic, milder aphasia and individuals within a similar age range (roughly 50–70 years). While the peer reviewed publication from Sather et al. (2017) does not discuss compliance in the nine individuals with aphasia that participated in the experience sampling methodology to evaluate ‘flow states’ in their daily lives, close reading of Sather’s (2015) dissertation suggests varying though largely good compliance with a higher number of notifications, albeit over a shorter period of time. The Sather (2015) thesis reports using six randomized indicators per day across a 12-h continuous period across seven consecutive days (i.e., 42 total sampled experience attempts). Table 6 from the thesis suggests that there was a range of compliance (*M* = 80.42%, SD = 17.49%, min = 42.86%, max = 92.86%), slightly lower than is reported in our current sample (*M* ~ 95%) but still relatively high for experience sampling.

While the best practice for dynamic sampling of inner speech appears to be mixed methodology involving experience sampling (Hurlburt et al., 2021), thought listing and interviews (Racy et al., 2020), and surveys (Alderson-Day et al., 2018; Racy et al., 2020, 2022), these were complex procedures to undertake in people with aphasia. The low number of total dropouts and the high session completion of this study is encouraging, given that we attempted to employ all three methodologies (though report only on quantitative data here). Note, though, that exclusion parameters (primarily a certain score on auditory comprehension) precluded the inclusion of individuals with more severe aphasia. The implications of this, and future directions, are discussed more below.

Experience sampling relies on self-report, which can be biased or inaccurate, especially in a population with possible comprehension and self-monitoring deficits, as discussed in Fama and Turkeltaub (2020). To lessen the concerns about self-report, participants were excluded based on poor auditory comprehension, and multiple modalities of communication (e.g., visual, written, auditory, gesture) were used throughout the study along with regular comprehension checks. Note, too, that the current samples presented with relatively little anosognosia, which likely reflects that the majority of the sample had intact self-monitoring as it related to monitoring of their own language impairments. The self-report methods used here were chosen to increase ecological validity and ability to measure contents of inner speech (Brinthaupt and Morin, 2023), while acknowledging the potential for inaccuracy. To mitigate potential biases known to occur during subjective reporting, such as the desire to please the experimenter [i.e., good-subject effect (Nichols and Maner, 2008)] experimenter JA reiterated the need for an accurate representation of what happens in daily life and encouraged honest reporting of the participant’s experience in that moment. Since participants met with JA a total of four times throughout the study (Introductory session, then three interviews), this was reiterated often. Future studies should be aware that revisiting the purposes of the study, and the procedures, are likely good practice for this type of methodology when collaborating with participants who have aphasia.

Another valuable takeaway of the current study was that the virtual and remote modality of meetings [i.e., introductory meeting, interviews (not described here), experience sampling, and questionnaires] was feasible for most participants. A small subsample elected to use a paper packet for the study, but all individuals employed some form of technology for the experience sampling procedure. Most individuals used their smartphone during the study, with only four of the 23 participants opting to use the paper packet. Those four, notably, differed in age range (50 years, 61 years, 71 years, 75 years), sex (two male, two female), education (14 years, 18 years, 20 years, 22 years), and prior occupation (art therapist, teacher, dentist, unknown/retired). It may have been a personal preference, but overall, use of technology (i.e., at least twice a day per experience sampling protocol) does not appear to be a barrier to participating.

### Exploratory question 2: frequency of inner speech use

The participants in this study reported experiencing inner speech often (nearly 80% of the sampled instances). Prior research in young adults have reported wide variation in inner speech experience, ranging from 0–75% (Heavey and Hurlburt, 2008; Brinthaupt et al., 2009; Heavey et al., 2019). It should also be noted that the pseudo-random prompt intervals in the current study may have led to heightened awareness around the inner speech experience, which may also help explain the larger proportion of study participants who experienced inner speech at the time of the notification. Although there could be multiple reasons for the differences in reported frequency, such as the varying methods (e.g., number of prompts given during experience sampling, number of total experiences sampled), this preliminary study suggests that people with chronic aphasia may experience inner speech relatively frequently in daily life.

Other studies broadly support that inner speech is available to individuals with chronic aphasia, and have used varying methods, such as asking individuals if they “can say the word in their head and it sounds correct” during object naming (Fama et al., 2019a,b; Fama and Turkeltaub, 2020) and if individuals with aphasia can successfully complete silent rhyme and homophone judgments (Geva et al., 2011a; Stark et al., 2017). Empirical research in adults without brain injury supports different cognitive and neural processes for spontaneously elicited inner speech (versus non-spontaneous inner speech) (what is being done in the experimental, word-level tasks) (Hurlburt et al., 2016; Grandchamp et al., 2019; Pratts et al., 2023). That is, spontaneous inner speech appears to not require as much ‘linguistic’ brain as non-spontaneous inner speech – likely because the spontaneous experience of inner speech draws upon semantic and lexical resources but perhaps fewer phonological and pre-motor resources – which means that individuals with damage to certain language regions of the brain may have heightened capacity for or awareness of spontaneous inner speech but not necessarily non-spontaneous inner speech. In the ConDialInt model (Grandchamp et al., 2019), this may reflect the fact that daily life inner speech experiences are likely more “condensed” than the experimental tasks probing word-level inner speech through rhyming and silent picture naming, which both require that the person have access to detailed phonological information. Despite the potentially different underlying cognitive and neural processes, the convergence of evidence from experimental and spontaneous (or naturalistic) tasks evaluating inner speech in aphasia appears to support that inner speech can be available to a person even in the context of a language impairment due to brain injury.

### Exploratory question 3: relationship between inner speech and demographic and other neuropsychological data

Exploratory analyses suggested that, in our sample, there was not a significant relationship identified between sex, age, or years post-stroke with inner speech. Note that this is a preliminary study with a small sample size, and as the power values included in the results stipulate, most correlations had low power. Low statistical power can occur because of small sample size, small effects, or both. In this case, there were no statistically significant correlations even when underpowered, suggesting that a much larger sample size would be needed to measure potentially very small effects (if indeed they exist).

A study employing the Varieties of Inner Speech Questionnaire suggested a higher rate of inner speech experiences that involved evaluation/criticalness in women (Alderson-Day et al., 2018), though their sample had 75.5% females. Largely, though, the impact of sex or gender on inner speech remains under evaluated (Morin and Racy, 2022). In the current study, we did not identify a compelling relationship between sex and inner speech in daily life.

Another exploratory area of interest was the impact of inner speech on age. Beyond the appearance and awareness of inner speech later in the childhood years (Flavell et al., 1997; Geva and Fernyhough, 2019), very little research has evaluated the impact of aging (adolescence, young adult, older adult) on inner speech frequency or its themes. In the sample of adults included in our study, which ranged from age 38 to age 75, a significant relationship of age with inner speech frequency or themes was not found. A medium-strong correlation was identified for the number of themes experienced but this effect was notably underpowered. If replicated in a larger sample, this negative relationship may suggest a relationship between increasing age with decreasing inner speech themes. A study on mind-wandering, which is not quite the same thing as inner speech but may comprise aspects of inner speech, suggested that the frequency of mind-wandering decreases with age in neurotypical aging, with no difference in the decrease due to age in individuals experiencing Alzheimer’s Disease (Gyurkovics et al., 2018). The authors offer the explanation that mind-wandering is a resource-dependent process and cognitive resources decline with age. If this were applied to our study’s findings, it might suggest that decreased cognitive resources result in fewer inner speech themes arising, though other factors are almost certainly also at play. For example, an increase in leisure time during retirement may decrease the need to use inner speech for certain reasons (e.g., problem solving) compared to younger adults. A much larger sample size would be needed to confirm any effect of age on inner speech.

We explored the relationship between chronicity – the number of years living with stroke consequences – and inner speech. Several anecdotal autobiographical accounts from stroke survivors who developed aphasia discuss the acute loss of inner speech, followed by a return of inner speech throughout their recovery process (Taylor, 2009; Marks, 2018). Observational evidence suggest that, in brain damaged patients who eventually recover from their trauma, self-awareness often returns in parallel with inner speech (Ojemann et al., 1996). Beyond recovery of inner speech during the acute phase, which is usually defined as the stage of spontaneous recovery during which most brain healing occurs (typically the first three months post-injury), little is known about inner speech changes in the chronic stage. Our study appears to not support a significant relationship between increased chronicity and inner speech frequency or themes in a sample of adults with a wide chronicity range (under one year to 25 years post-stroke). Therefore, years of living with chronic stroke symptoms may not be the most salient variable for describing any potential changes observed in inner speech.

Finally, we examined the extent to which anosognosia (lack of insight specific to language impairment) related to inner speech, given inner speech’s strong relationship with self-monitoring (Morin, 2011a,b). Documentation of aphasic anosognosia can be traced back to Wernicke but has only recently been quantified using standard assessments (Wernicke, 1874; Cocchini et al., 2010; Kertesz, 2010). Therefore, it would not be surprising to find that experience of inner speech is negatively related to anosognosia – that is, people with probable anosognosia may report experiencing fewer instances of inner speech. Another thought could be that those with anosognosia may not reliably self-report inner speech function, and/or may over-estimate how much they use inner speech. However, to our knowledge, no study has empirically related presence of anosognosia with inner speech experience in aphasia. Our study did not find strong evidence that the amount of anosognosia, or lack of insight, related to inner speech frequency or themes. It may be because only two individuals in the current study demonstrated a discrepancy score that the original (Cocchini et al., 2010) study deemed “anosognostic,” i.e., > = a discrepancy of 13.1. That is, there may not have been a sufficient sample of individuals experiencing anosognosia to reliably identify any relationship with inner speech. This may once again be due to a relatively mild presentation of aphasia in the majority of the participants in this study, who are less likely to present with anosognosia (Kertesz, 2010), but it should be noted that others have not found a direct relationship between anosognosia and aphasia severity (Weinstein and Kahn, 1950, 1955). The neural and cognitive mechanisms specific to anosognosia of language impairment in aphasia remain unexplained (Kertesz, 2010), though there is likely some relation to a self-monitoring mechanism. Some scholars have postulated a language-related self-monitoring mechanism situated in auditory comprehension (Roelofs, 2020) (which is leant some credence by the documentation of individuals with Wernicke’s aphasia as often presenting with anosognosia; Wernicke, 1874), while others have argued for a more generalized self-monitoring system less intimately tied to the language system (Nozari et al., 2011). While the current study does not directly support either claim, some level of self-monitoring seems important for understanding the experience of inner speech. This may also be why some individuals without brain damage say that they lack inner speech (Hurlburt et al., 2021), i.e., self-monitoring may be an individual difference that helps explain the inner speech phenomenon. This is a potent area of future study, in aphasia and in adults without brain damage.

It would be remiss to not comment on the research suggesting a relationship between meta-cognitive and executive function capacities with inner speech, and how that may play a role into the perception of inner speech by persons with aphasia who may have impairments in these areas. Philosopher Langland-Hassan theorized that, whilst theorists have linked inner speech with metacognition (thinking about one’s thinking), it does not appear that inner speech has a clear correlation or reason for being related to metacognitive capacities given that metacognition is not thought to be particularly related to inner speech’s linguistic functions, its sensory characteristics, or its consciousness (Langland-Hassan, 2014). Indeed, further work from his group suggested that inner speech impairment (as measured by a highly linguistic task, which is the covert judgment of rhyming pairs) was likely not related to metacognitive self-assessments in general (Langland-Hassan et al., 2017). Inner speech’s relationship with executive function has also been explored in neurotypical adults, suggesting that disruption of inner speech (through things like articulatory suppression) results in problem solving impairments (Wallace et al., 2017). Whilst executive function impairments are not pervasive in aphasia, they can be present (Purdy, 2002; Fridriksson et al., 2006; Mayer et al., 2016; Dutta et al., 2022). No study, to our knowledge, has yet examined the relationship of executive dysfunction and inner speech in aphasia. This question is especially interesting in light of theories suggesting that speech self-monitoring relies on domain-general executive function ability (Nozari et al., 2011). This is a clear future research direction that should be explored, especially to elucidate the directionality of inner speech and executive dysfunction. For example, it may be the case that inner speech is required for higher executive function capacity and that, when executive function is impaired, inner speech is not impaired but instead simply cannot be leveraged to its full extent.

#### Cases of less frequent inner speech

In this exploratory pilot study, most of the people with aphasia reported frequent use of inner speech, but there were two participants who reported inner speech less often. These participants had unique clinical profiles, including moderate motor speech deficits for one participant (Participant 20) and anosognosia (lack of awareness of language deficits) and low compliance for another participant (Participant 30). These cases motivate future work on the topic, specifically investigating speech motor programming, anosognosia, and inner speech with a greater variety in the clinical profiles of the participants. Links may elucidate the nature of inner speech and its place in lexical retrieval, speech production, and self-monitoring.

Participant 30, who was classified as having anosognosia, may have reported less frequent inner speech due to a unique inner experience. During interviews, this participant reported a version of “condensed” inner speech: he did not always *hear* or *say* the words in his head (Heavey and Hurlburt, 2008; Hurlburt et al., 2016), but rather *saw* the words, as if on a teleprompter. That is, phonological properties may not have been present for his experience of inner speech, but some access to visual representations of lexical-semantics appeared available. Uncovering individual differences in the phenomenon of inner speech experience is crucial for understanding how inner experience relates to behavior, as individual differences in its properties may change how and to what extent a person uses it (Roebuck and Lupyan, 2020; Lupyan et al., 2023).

### Hypothesis 1: themes of inner speech use

A major purpose of leveraging the General Inner Speech Questionnaire was to evaluate the themes of inner speech use. The use of the General Inner Speech Questionnaire at the end of each week of experience sampling was intentional, given evidence that experience sampling tends to result in within-subject and across-subject patterns and differences (Hurlburt and Akhter, 2006). That is, across Hurlburt’s studies employing descriptive experience sampling, he found that the more participants were asked to be consciously aware of inner speech (through the experience sampling notifications), the more they tended to recall about inner speech and be “conscious” of inner speech. Therefore, it was our thought to have the General Inner Speech Questionnaire come at the end of each week of experience sampling, where there would be a probability of heightened awareness as to the inner speech experiences across that week.

In this preliminary study, participants with aphasia in the current study experienced similar themes of inner speech as found across several studies in young adults without brain injury. Indeed, Racy et al. (2020) and Morin and Racy (2022) summarize that the most common inner speech experiences found in over 1,000 undergraduate volunteers included content themes (negative emotions) and functions (to problem solve, plan, control emotions, and self-motivate). In the current study, functions were the most endorsed reasons for using inner speech, with solving problems, thinking critically, planning, rehearsing ahead of time, and concentrating as some of the most shared experiences. Content experiences were also prominent, with negative emotions, preferences, “what I want to do,” and food/what I want to eat as experienced by many within the sample. Therefore, our findings suggest some commonality to inner speech experience in aphasia compared to individuals without brain injury.

There is a distinct lack of data about the inner speech experiences of older adults. It may be that some of the experiences found in the current study’s sample are more reflective of being an older than a younger adult, e.g., we showed that 56.52% of the sample said (at least once) that their inner speech involved health. The same could be said about using inner speech during remembering, and perhaps also with coping (with 56.52% of our participants using inner speech to cope at least once during the study), the latter of which is a much rarer use of inner speech in adults (Racy et al., 2020). A clear future direction is collection of inner speech experiences in the older adult population.

While there is a large benefit to comparing themes between adults with aphasia and neurotypical data collected previously using the restricted list of themes available on the General Inner Speech Questionnaire, there are also drawbacks to this approach. For example, it may be that persons with aphasia endorse different themes than those on the General Inner Speech Questionnaire modified version. Our team did anticipate this, collecting thought listing data during each experience sampling session and at the end of each General Inner Speech Questionnaire administration (specifically, we asked: “What else do you talk to yourself about? Use this space to write or draw”). However, team capacity has not yet enabled us to aggregate and analyze that qualitative data. Therefore, the extent to which adults with aphasia endorse *different* themes from those in the General Inner Speech Questionnaire is the subject of future work from our group and will extend the findings presented here.

The ratings of confidence in their inner speech themes also warrant consideration here. It has been shown previously that neurotypical young adults responding to the General Inner Speech Questionnaire are generally confident in their accurate recall of inner speech experiences across the past week (Racy et al., 2020), and the present study replicated that in a small sample of persons with aphasia. However, it bears consideration that persons with aphasia may have varying judgments of confidence. There is a body of literature that suggests that persons with aphasia may not be confident in their overall communicative abilities (Babbitt and Cherney, 2010), and it bears questioning whether this extends to confidence in their perception of inner speech. Whilst it was not the goal of the current study to probe this, this is a consideration that warrants future consideration, because heterogeneity in confidence – if this study were to be extended to include a more diverse representation of persons with aphasia—may be found, and this could be used to examine if and how themes and frequency of inner speech relate to perception of each person’s confidence in reporting inner speech.

We explored the similarity of inner speech experiences across weeks, with no directional hypothesis attached to this. The rationale behind this exploratory investigation was that, because of the relationship of inner speech with context (i.e., it shifts according to scenario, mood, need, etc.) (Alderson-Day et al., 2018; Heavey et al., 2019; Racy et al., 2020), there may be corresponding fluctuations across inner speech items across the three administrations/weeks. Indeed, it is expected that life circumstances will vary across participants. Despite this rationale, findings from the current study did not suggest much shift in inner speech themes (total number or type) over the course of three weeks. This consistency (or lack of significant difference) may be early evidence for short-term reliability of administering the General Inner Speech Questionnaire in this sample. Longer studies (i.e., over the course of several months or even years) may be more likely to demonstrate changes in inner speech due to the higher potential of significant life or context changes occurring then. We also did not collect information about any significant life events that may have occurred during the study, and future studies should collect this to evaluate the extent to which these life events impact inner speech.

### Hypothesis 2: inner speech’s relationship with aphasia severity

The hypothesis was a null hypothesis (that aphasia severity would not be related to inner speech) and statistics failed to reject the null hypothesis. This is an important finding because it suggests that future research should evaluate the extent to which individuals with more severe aphasia feel that they experience inner speech. If inner speech was only an impoverished version of overt speech, people with more severe aphasia should experience inner speech comparatively less than those with milder aphasia. Note that there were no individuals with severe aphasia in the current sample, precluding a more conclusive interpretation of the impact of more severe aphasia on inner speech. Failing to reject the null hypothesis in the current study motivates future research into the role of inner speech, such as indicating which items are most stimulable in therapy (Hayward et al., 2016) and identifying the root cause of someone’s anomia (Fama et al., 2019b; Fama and Turkeltaub, 2020).

Prior work evaluating more experimentally-obtained inner speech, such as subjective inner speech experience during picture naming or rhyming, has suggested that some adults with severe non-fluent aphasia have inner speech to a degree, and that their inner speech is often preserved more than their overt speech (Geva et al., 2011a; Stark et al., 2017; Fama et al., 2019a). However, spontaneous inner speech occurring in daily life in individuals with severe aphasia, inclusive of those experiencing more impairments related to auditory comprehension, is a clear future direction of research. It is likely that more accommodations need to be made to methodology and procedures to encourage participation from this group, and developing this study is a prime opportunity to employ participatory design by partnering with individuals with severe aphasia to co-design the procedures. It may also be the case that, by nature of the type of strict procedural requirements of experience sampling, that a necessary cut-off of auditory comprehension abilities must be employed to ensure study validity, and that we, as a field, may be unable to understand the daily experience of inner speech in individuals with more severe auditory comprehension or self-monitoring impairments using this type of procedure.

## Conclusion

For what we believe is the first time, we dynamically evaluated self-report of inner speech experiences in the daily lives of adults with chronic aphasia. This preliminary study had low attrition and high compliance, establishing that participants could understand and execute this method, despite it being time intensive. Most participants reported experiencing inner speech, and there was a general pattern in the themes of inner speech that were common amongst the group, including using inner speech to remember, plan and motivate oneself. Demographic variables (year post-stroke, age, sex), aphasia severity, and insight into language impairment were not found to have a significant relationship with inner speech frequency or the number of themes explored using inner speech.

### Broader implications and clinical application

We envision several broad implications and clinical applications of assessing inner speech in daily life in adults with aphasia. By collecting inner speech data from adults with aphasia, clinicians (such as speech-language pathologists and psychologists) may choose goals that take into account that people with aphasia are doing certain tasks “in their heads” using inner speech (e.g., planning, rehearsing a conversation), meaning that (1) they are likely salient to the individual with aphasia, and (2) stimulating verbal language around those tasks during therapy may be particularly beneficial, given that inner speech has been demonstrated to relate to overt language recovery with therapy (Hayward et al., 2016). We envision that researchers, speech-language pathologists, and mental health professionals can leverage the types of procedures used in this study to better understand changes in emotional states and psychosocial health without relying heavily on verbal production. Most participants used inner speech to talk about negative emotions, in order to regulate emotions, and in order to motivate themselves. Because of the relative strength of inner speech compared to overt speech in many people with aphasia (Geva et al., 2011a; Fama et al., 2017; Stark et al., 2017), much of a person with aphasia’s language may be taking place inside their heads. This is especially likely in cases of comorbid dysarthria and apraxia of speech, in which overt motor speech is impacted. Specifically for apraxia of speech, which is a disorder of motor planning and programming, evidence of successful inner speech indicates that internal retrieval and modeling can be intact without successful overt word production (Fama et al., 2019a). In the current study, less emphasis was placed on requiring inner speech to have all correct phonetic information, but in general, successful inner speech requires accurate lexical retrieval and access to semantic information. Clinicians should be aware of this possibility and recognize that skills such as “rehearsing ahead of time” may be more common in inner speech post-stroke and could be leveraged during therapy (e.g., practicing scripts using inner and overt speech; asking if the person has inner speech for items or phrases post-therapy to gauge improvement in not only overt speech but also inner speech over time).

As discussed above, anecdotal evidence from lived experience suggests that inner speech may disappear in the acute stage of aphasia and recover over time, perhaps in tandem with or slightly ahead of the rest of language (Taylor, 2008; Marks, 2018; Anbar, 2022). This suggests that clinicians in the acute and inpatient settings may want to monitor changes in inner speech as reflective of recovery, just as they would measure expressive and receptive language changes. Along with the outcome measures that are generally collected, inner speech can be added to the unique profile of communicative abilities for the clinician’s patients with aphasia.

Finally, aphasia recovery does not singularly involve language improvement. Indeed, because individuals with aphasia become more socially isolated (Code, 2003; Dalemans et al., 2010) and experience a variety of other challenges (e.g., depression, Leeds et al., 2004; Pompon et al., 2022), recovery of language and communication occurs more holistically. The Life Participation Approach to Aphasia suggests focusing on the person with aphasia’s experience across a variety of domains, including personal factors, environment, and activities/participation (Chapey et al., 2018; Kagan, 2020). Measuring and leveraging inner speech in daily life may be a unique way to holistically understand aphasia recovery.

## Limitations and future directions

We employed pseudo-random interval sampling during the study, which was chosen to increase predictability of prompts (e.g., within the same hour per day), reduce prompt fatigue, and increase compliance (Hektner et al., 2007). The pseudo-random, semi-predictable signal times carry strengths and weaknesses. For example, when people know that they will be notified for a short survey every day between 9-10 AM and 4-5 PM, they tend to adjust to the interruption, e.g., they may plan to not be driving or be in a quiet spot in order to accurately and quickly respond (Hektner et al., 2007). A strength of predictability is that people tend to not find the alerts intrusive, so compliance will be high and missing data will be low – this is indeed what we found in our study. A weakness, though, is that people may rearrange their activities in order to “fit in” with the data collection regime, e.g., avoid scheduling things during those times. This may result in heightened “awareness” of inner speech, e.g., in the case of our sample, thus creating a slightly artificially high number of “in the moment” yes responses. However, given that individuals with aphasia have many unmovable commitments (e.g., support groups, therapy), we felt that the strengths of pseudo-random design outweighed the weaknesses. This design choice poses a limitation for comparison of our findings to other descriptive experience sampling studies of inner speech (e.g., Hurlburt and colleagues’ work in young adults), which involved random sampling as well as more samples per day (typically around ~six notifications per day, though the total number of notifications was similar to what was employed in our study). Future studies should replicate the findings from the current study as well as empirically explore the extent to which participants with aphasia are amenable, and reliable, in studies employing a variety of beep/prompt intervals (e.g., up to six per day) and study durations (e.g., days, weeks).

As acknowledged in the method and results, there was one option on the General Inner Speech Questionnaire which was included on the Qualtrics survey but not on the paper packet (“Replay past conversations” was listed twice rather than including “Listen to my own voice”). This was an experimenter error and results must be interpreted as such because of the missing data. However, given that replaying past conversations was reported relatively frequently across all weeks (>60% of participants) and listening to my own voice items was reported relatively infrequently across all weeks (<20% of participants), the missing data may be interpreted as largely negligible.

This study’s sample did not include any participants with severe aphasia, which may affect the relationship with aphasia severity and reports of inner speech, as their experience is not captured here. Additionally, comparisons to prior literature are limited due to the age difference between the participants, as frequency and contents of inner speech may change throughout the lifespan regardless of brain injury. Future work involving individuals with more severe aphasia and anosognosia is the logical next step in validating the impact of each on inner speech, along with comparisons to age-matched adults without brain injury or with other neurogenic communication disorders (e.g., traumatic brain injury, right hemisphere damage, or cognitive-communication impairments). Hurlburt and colleagues’ body of work also suggests that the validity and specificity of descriptive experience sampling improves with training and practice. Since this study established early evidence for feasibility of the use of descriptive experience sampling in a population of adults with aphasia, future work should evaluate the extent to which increased training (such as a longer study, or an extended introductory period) complements the results presented here.

Finally, it is of note that the General Inner Speech Questionnaire was modified based on consultations with an expert in the field of inner speech research, Alain Morin. A clear next step is to engage with persons with aphasia to further refine the scale, ensure its accessibility, and ensure its specificity to living with an acquired language impairment.

## Data availability statement

The datasets presented in this study can be found in online repositories. The names of the repository/repositories and accession number(s) can be found at: https://osf.io/b6vxk/.

## Ethics statement

The studies involving humans were approved by Indiana University Institutional Review Board (study #10549). The study was conducted in accordance with the local legislation and institutional requirements. The ethics committee/institutional review board waived the requirement of written informed consent for participation from the participants or the participants’ legal guardians/next of kin because verbal consent was enabled given procedures occurred during COVID-19.

## Author contributions

JA: Data curation, Formal analysis, Investigation, Methodology, Project administration, Supervision, Validation, Visualization, Writing – original draft. TH: Data curation, Formal analysis, Investigation, Writing – review & editing. BS: Conceptualization, Data curation, Formal analysis, Funding acquisition, Investigation, Methodology, Project administration, Resources, Supervision, Validation, Visualization, Writing – original draft, Writing – review & editing.

## Footnotes

^1^ https://osf.io/qf45v

^2^ https://osf.io/b6vxk/
