# Supplementary material for: Inner speech in the daily lives of people with aphasia
Source: Front Psychol. 2024 Mar 21;15:1335425. doi: 10.3389/fpsyg.2024.1335425 (PMC10991845; doi:10.3389/fpsyg.2024.1335425)
Supplement: Supplementary file 1 [file Data_Sheet_1.docx]

Supplementary File 1 (S1): The adapted General Inner Speech Questionnaire, complete with pictures. It includes sections on the contents (“I talk to myself about…”), functions (“I talk to myself in order to…”, and activities (“I talk to myself when…”) of inner speech use. It can be found at <https://osf.io/b6vxk/> under “Files.”
