# Supplementary material for: Inner speech in the daily lives of people with aphasia
Source: Front Psychol. 2024 Mar 21;15:1335425. doi: 10.3389/fpsyg.2024.1335425 (PMC10991845; doi:10.3389/fpsyg.2024.1335425)
Supplement: Supplementary file 2 [file Table_1.docx]

# Supplementary Materials

Supplementary Table 1: Removed items from the General Inner Speech Questionnaire (middle column) to form the adapted version used in the current study (right column), organized by General Inner Speech Questionnaire section (left column). GISQ = General Inner Speech Questionnaire.

| Section | Items included in original GISQ but not included in Adapted GISQ | Items included in both Original and Adapted GISQ |
| --- | --- | --- |
| I talk to myself about (content) | - Behavior - Sleep - Beliefs - Music - Physical activity - Hypothetical situations - Family - Friends - Education - Appearance - Motives - Work - Ideas - Immediate environment - Intimate partner | - Positive emotions - Negative emotions - What others think about me* (modified wording) - Errands - Who I am - Bodily sensations - Preferences - People around me - Past events - What I want to do* (modified wording) - Food / what to eat - Current events - Health - Financial situation - Future - Relationships |
| I talk to myself in order to (function) | None removed | - Replay past conversations - Solve problems - Motivate myself - Control emotions - Think critically - Plan - Rehearse ahead of time - Avoid saying out loud what I want to say - Concentrate - Listen to my own voice - Cope |
| I talk to myself when (activities) | - Ruminating - Alone/bored - Studying - Performing hygiene - At school - At work - Walking - Mind wandering - Orienting myself - Taking transit | - Meeting people - Reading* (modified wording) - I want to rely on myself - Remembering - Driving |
